# Optimizing Complex Kinetics Experiments Using Least-Squares Methods

**DOI:** 10.6028/jres.098.013

**Published:** 1993

**Authors:** A. Fahr, W. Braun, M. J. Kurylo

**Affiliations:** National Institute of Standards and Technology, Gaithersburg, MD 20899-0001

**Keywords:** CH_3_O_3_, computer modeling, complex gas phase kinetics, free radicals, HO_2_, least squares, optimization, simulated data

## Abstract

Complex kinetic problems are generally modeled employing numerical integration routines. Our kinetics modeling program, Acuchem, has been modified to fit rate constants and absorption coefficients generically to real or synthesized “laboratory data” via a least-squares iterative procedure written for personal computers. To test the model and method of analysis the self- and cross-combination reactions of HO_2_ and CH_3_O_2_ radicals of importance in atmospheric chemistry are examined. These radicals as well as other species absorb ultraviolet radiation. The resultant absorption signal is measured in the laboratory and compared with a modeled signal to obtain the best-fit to various kinetic parameters. The modified program generates synthetic data with added random noise. An analysis of the synthetic data leads to an optimization of the experimental design and best-values for certain rate constants and absorption coefficients.

## 1. Introduction

Many rate constant determinations, particularly for radical-radical reactions, are obtained in complex kinetic systems in which more than one rate process and more than two species concentrations are involved. The kinetic processes can be first or second order or can be mixed. In such cases, an analytic relationship involving species concentrations and rate constants is often impossible to attain and numerical integration is required. If the measurements involve optical absorption, for example, an added complication of contributions to the signal from more than one species may arise. There are a number of modeling programs available to handle such complex systems but most are written for main-frame computers and few are capable of optimizing the fit of a given model to laboratory data by simultaneously adjusting a number of parameters [1]. To obtain a “best-fit” of the data to the kinetic parameters the least squares procedure can be applied. While there is in principle only one least squares method [2,3], its application requires consideration of the conditional constraints relevant to each problem. These are sometimes referred to as the condition or adjustment equation(s).

We describe in this paper the application of a computer program to a least squares fit that is applicable to any mechanism. However, the condition equations may be incomplete and the procedure for analyzing the fit of the parameters to the data should be examined carefully by the user. The kinetic modeling computer program, Acuchem [4] is now widely used. It has been modified in the present work to: 1) perform “Monte Carlo” simulations, and 2) to analyze synthetic data (with noise) via the least squares method and to yield a best fit to various kinetic parameters within the constraints of the experiment. The program can also serve for the analysis of laboratory data as well an aid in the optimization of experimental design.

The full program capabilities can be demonstrated through the application to the analysis of an important laboratory kinetics problem − which will be done in this paper. Here, the application of the program will be emphasized rather than its detailed construction. The details of the program will be published in near future. However, an interested reader wishing to implement methodology described here could request the program from authors.

## 2. Procedure

The Acuchem modeling program requires an input file containing a kinetic mechanism with initialized rate constants and initial species concentrations. An output file is generated which lists the concentrations of all of the species on a preselected time grid, and can be displayed as a user-directed graph. We have written an additional program that converts the Acuchem output file into a synthetic “laboratory” data file. The name of each species is printed separately and the user can enter the contribution of each species in the composite “experimental” absorption signal. In normal practice, the synthetic data would be used to determine the sensitivity of the parameter(s) whose values are being sought to other measured variables.

A new analysis program called Acufit has been devised. It reads the Acuchem input file, assumed to contain the correct mechanism or “model,” and then either the synthetic “laboratory” or real laboratory data. This is fitted to the “model” in a prescribed way. Both rate constants and absorption coefficients (representing the contribution of each species to the signal) can be adjusted. An iterative adjustment procedure is employed. These problems are inherently nonlinear and one adjustment around initial “first guess values” (by taking only first derivatives) is usually not sufficient. Of course first guess values are necessary and, as long as they are not too divergent from the “correct” values, the iterations will converge (if a solution is possible, see below). Adjustments are made via solution of the “normal equations.” Because the variance-covariance matrix is readily available, the quality of the fit can be assessed. Solving for all possible parameters in the system is usually not possible in that multiple (degenerate or near-degenerate) solutions can often fit the data equally well. Programs similar to the one described here are not fully automatic because operational decisions must be made. The possible variations in the analysis method are explored.

## 3. Mechanism (Model)

The specific kinetic problem we investigate here consists of determining the cross-combination rate constant for two radicals of atmospheric interest, HO_2_ and CH_3_O_2_. Additional details of this system can be found elsewhere [5,6]. The experiments are complicated by overlapping absorptions from HO_2_ and CH_3_O_2_, uncertainties in absorption cross sections, and the inability to perform the experiment under first-order conditions. The objective of this paper is not resolving the disagreement concerning the correct values for absorption cross sections or rate constant for HO_2_ + CH_3_O_2_ but to demonstrate the application, capabilities, and limitations of our current kinetic modeling program. To do so, we have chosen the initial conditions and results of earlier studies of this system in our laboratory. Primarily, because conditions of other studies are not very clearly outlined in the literature [5,6].

There are three dominant reactions, two combination reactions and the cross-combination reaction. The initial radical concentrations (or rather their ratio) are in the range of previously used experimental values [5,6]. The units below in Eqs. (1) and (2) are cm^3^ molecule^−1^ s^−1^ for *k_x_*, molecules cm^−3^ for concentrations, nanometers for λ, and 10^−20^ cm^2^ (base e) for absorption coefficient, ϵ*_x_*.

$$\begin{array}{ll} {\mathrm{HO}}_{2}+{\mathrm{HO}}_{2}\to H_{2}O_{2}+O_{2}, & k_{1}=1.86\times 10^{-12}\\ {\mathrm{CH}}_{3}O_{2}+{\mathrm{CH}}_{3}O_{2}\to \text{products}, & k_{2}=3.60\times 10^{-13}\\ {\mathrm{HO}}_{2}+{\mathrm{CH}}_{3}O_{2}\to {\mathrm{CH}}_{3}O_{2}{H+O}_{2}, & k_{3}=2.90\times 10^{-12}\\ {\left[{\mathrm{HO}}_{2}\right]}_{0}+{\left[{\mathrm{CH}}_{3}O_{2}\right]}_{0}=10.8\times 10^{13}, & \lambda \left(215\right)\\ {\left[{\mathrm{HO}}_{2}\right]}_{0}+{\left[{\mathrm{CH}}_{3}O_{2}\right]}_{0}=11.8\times 10^{13}, & \lambda \left(250\right) \end{array}$$ (1)

The parameters listed are used to synthesize a good surrogate to the “real” laboratory data, which can be used for test purposes. The objective here is to assess the optimum conditions for obtaining a best fit to the cross-combination rate constant *k*_3_. The rate constants *k*_1_ and *k*_2_ and the ϵ’s are best obtained in single component systems [7,8,9,10] containing only HO_2_ or CH_3_O_2_.

Any complex experiment of this kind entails conditions that are characteristic of the experimental design. Those that apply to an earlier study from this laboratory [5] are given in Eq. (2).

$$\begin{array}{l} \epsilon {\mathrm{HO}}_{2}\times {\left[{\mathrm{HO}}_{2}\right]}_{0}+\epsilon {\mathrm{CH}}_{3}O_{2}\times {\left[{\mathrm{CH}}_{3}O_{2}\right]}_{0}=\\ \;3.73\times 10^{-4},\;\lambda \left(215\right)\\ \epsilon {\mathrm{HO}}_{2}\times {\left[{\mathrm{HO}}_{2}\right]}_{0}+\epsilon {\mathrm{CH}}_{3}O_{2}\times {\left[{\mathrm{CH}}_{3}O_{2}\right]}_{0}=\\ \;3.39\times 10^{-4},\;\lambda \left(250\right) \end{array}$$ (2)

The conditions listed in Eq. (2) arise because, i) the sum of the initial concentrations, [HO_2_]_0_ and [CH_3_O_2_]_0_, results from the titration of known number of precursor Cl atoms, and ii) for weak absorptions the measured absorption signal is linear in the sum of the product of the absorption coefficient and the concentration (and path length) for each respective species. The numbers given under Eq. (2) must always be consistent with those listed under Eq. (1). Also, for convenience, the program requires that the *t* = 0 concentrations be initialized and cannot be adjusted by the program. In the present work only dimensionless units are employed. This does not influence the analysis of errors.

## 4. Results of Experiments Performed at 250 nm

We have applied the Acufit program to the model defined by Eqs. (1) and (2) with a view towards: i) identifying the number of parameters that have to be fixed under various noise/signal (*n/s*) conditions; ii) specifying different parametric values (in groups) in order to identify those producing the least bias in the analysis of *k*_3_; Hi) fixing all parameters with the exception of *k*_3_, the cross-combination rate constant, in order to provide an independent check on the error analysis obtained in a previous experimental study; and iv) identifying initial concentration ratios of reactants which produces the least bias on *k*_3_ given variations of known parameters within their expected uncertainty. Results are presented in tabular and graphic form demonstrating the effects of these changes in the method of analysis.

### 4.1. Fixed Parameters, at “Perfect” Values

As stated earlier Acufit requires as input: i) a synthesized or a real laboratory data file, and ii) the Acuchem (model) input data file. In the example we used here the synthesized file was constructed using the values of *k*_1_, *k*_2_, *k*_3_, *ϵ*HO_2_, *ϵ*CH_3_O_2_, *ϵ*H_2_O_2_, *ϵ*CH_3_O_2_H, [HO_2_]_0_, and [CH_3_O_2_]_0_, given under Eq. (1) for λ =250 nm. The Acuchem (model) input file contains all of the information listed under Eq. (1), i.e., the mechanism, the initial concentrations (always assumed to be known) and the rate constants, *k*_1_, *k*_2_, and *k*_3_ taken as initial first estimates. Acufit also requires specification of the parameters (*k*’s or *ϵ*’s) to be evaluated and their initial assignments. Parameters not to be evaluated (fixed) are held at their input values. Finally Acufit requests the value for the random (normally distributed) noise on the data and whether the noise distribution is to be always the same (unseeded) or randomly variable (seeded) from run to run. In the present study the “noise” or *σ* applied to the data is taken to be independent of the signal level, common for most absorption experiments.

Since the initial assignments for all of the parameters are similar to those used in the construction of the synthetic “laboratory” file, Acufit might return values very close to these, depending upon the entered level of noise. In most cases, however, the sums of residuals squared either increase with successive iterations or oscillate such that some of the adjusted rate constants or absorption coefficients become negative (the program returns negative *k*’s as zero). This means that this problem is not sufficiently constrained so that a unique solution can be found. If this occurs one or more parameters must be fixed at some prescribed input value. If that value is the *same* as used in the construction of the synthetic “laboratory data” file, it is called a “perfect” value. If it is “fixed” at a different value it is called “imperfect.” Only when “perfect” values are used for the *fixed* parameters will the analysis program return values for the *adjusted* parameters that are the same as those used in the construction of the synthetic file, i.e., within the precision given by the variance-covariance matrix. The precision, of course, depends on the noise level.

**Fig. 1:**
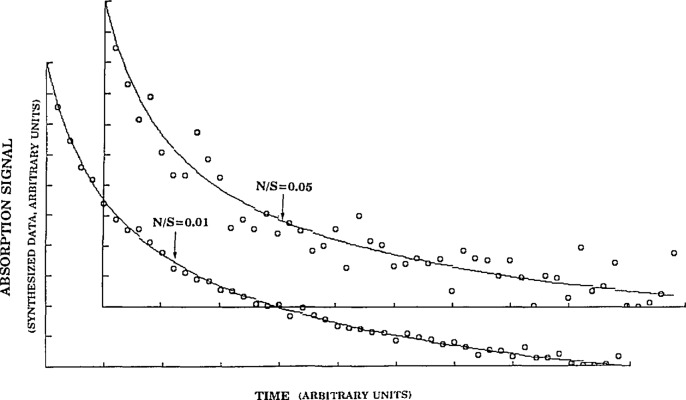
Best fit to the data when the *n/s* ratios are 0.01 lower and 0.05 upper (insets). Parameters employed in synthesis and analysis are given in column 1, Table 1.

Table 1 was constructed to illustrate variations in the method of analysis. Column 0 labels all parameters that affect the outcome of the best-fit of the data to the model. Values used in creating the “synthetic laboratory” file are listed in column 1. Subsequent columns list results of the analysis with error limits obtained from the variance-covariance matrix. If a cell contains the symbol *F*, the exact value for that parameter as given in column 1 is employed. If the value is fixed at a value different from that in column 1 it is so designated (for example *F* = 0). All columns involve analysis of the same “synthetic” laboratory data file. This file contains the same noise distribution (at the same or different level as specified in the first row). That is, in running the program the random number generator that produces the distribution is “not seeded.” Figure 1 shows the best-fit to the synthesized signal at 0.01 and 0.05 *n/s* levels. It is noted that in spite of the different intensities, the distribution with regard to the noise is the same. The best-fit to the synthesized data, in Fig. 1, was obtained by fixing all parameters at their “perfect” values, (see column 1, Table 1). Most laboratory experiments are performed at *n/s* levels between these two extremes. In comparing the synthesized signal to a real lab signal it is important that the *n/s* levels be properly compared particularly if dimensionless units are employed.

If a file is produced from a program in which the noise distribution is seeded, it is much the same as when a given experiment is re-run. Regardless of whether the noise distribution is seeded or not the error in fitting a parameter should usually fall within the error estimate given by the variance-covariance matrix. The error analysis may be checked by re-running in the seeded mode. Each experiment returns a slightly different value for the adjusted parameters and the standard deviation may be compared with the variance-covariance error estimates. Such checks demonstrate that the program performs properly.

For this mechanism or model, the program will not be able to adjust all parameters (Appendix A). There may be as many as nine parameters. It is unusual in such kinetic mechanisms for the adjusted parameters to be independent of one another. The variance-covariance matrix shows the degree of correlation or anti-correlation between them. Species concentrations are excluded from adjustment, a reasonable programming decision that can be relaxed at some later time. For this mechanism the maximum number of parameters that can be uniquely adjusted is four (Table 1). The number depends on the level of noise on the data.

To obtain the best fit to *k*_3_. the question arises as to whether those parameters which contain considerable error should be fixed. Table 1 provides an answer to the question for at least one case. The analysis listed under column 5 shows a poor value for *ϵ*HO_2_, the error being as large as the value of the parameter. The error obtained for *k*_3_ is ± 0.78. In column 6 the parameter for ϵHO_2_ is fixed at its “perfect” value, *ϵ*HO_2_ = 60. As a result a better value of *k*_3_ is obtained with an error of ±0.20. In this case, provided that *ϵ*HO_2_ is known well enough, it would clearly be better to fix it than search for it. If for example, *ϵ*HO_2_ were fixed at an “imperfect” value of 30 while maintaining all of the other entries in column 6, *k*_3_ = 3.2 ±0.22, would be obtained, a better value than that shown in column 5. We will explore in detail the effect of fixing parameters at “imperfect” values in the next section but emphasize here the well quoted rule that if a parameter is obtained with an error estimate comparable to its value it may be better to fix that parameter.

Table 1 shows, as expected, that the parameter of interest is obtained with better precision when a larger number of parameters are fixed. This advantage is offset by the fact that fixing parameters at “imperfect” values introduces a bias in the results. Table 1 shows further that the model is very insensitive to *ϵ*H_2_O_2_ and *ϵ*CH_3_O_2_H in that setting these absorption coefficients equal to 0 has little influence on the adjusted parameters (columns 7 and 8). Since all parameters cannot be adjusted simultaneously, the best strategy is to fix them at their “perfect” values. As shown in Table 1 the errors given by the variance-covariance matrix for the various parameters is closely linear in the *n/s* level (columns 9 and 10, as well as 13 and 14).

The main observations from the analyses listed in Table 1 are, i) a number of parameters must be fixed, ii) fixing some combination of parameters is superior to other combinations. Parameters that show the greatest error estimates should preferably be fixed, iii) the amenability of adjustment for certain parameters is a function of the noise level and initial conditions, and finally, iv) if all fixed parameters are entered at their “perfect” values the variance-covariance matrix error estimates are completely reliable.

### 4.2. Fixed Parameters at “Non-Perfect” Values

We now explore the consequence of not entering “perfect” values for the fixed parameters in the calculations. After all in an actual experiment, we do not usually know the exact value for a fixed parameter, only an average value with a plus and minus uncertainty. The conclusion is that fixing a parameter at a “non-perfect” value simply produces a bias on the values of the adjusted parameters yielded by the analysis. The strategy is to fix only those parameters causing the least bias on the parameter of interest, *k*_3_ in the present example. When biases are present, estimates of errors (of the adjusted parameters) obtained from the variance-covariance matrix are likely to be too small. Such biases are best investigated by means of the present procedure through the use of synthetic data. The biases can then be determined, and only when they are added to the variance-covariance error estimate is there an appreciation of the real error in such a system.

In developing Table 2 a few observations are noted. The fixed parameters were generally chosen ±10% from their “perfect” values, column 1. This produces biases that are nearly symmetric. Since the program does not provide for adjustment of initial concentrations, when either [HO_2_]_0_ or [CH_3_O_2_]_0_ is fixed at an “imperfect” value it is necessary to adjust one or both of the absorption coefficients, *ϵ*HO_2_ and *ϵ*CH_3_O_2_ so that the implicit constraint, [HO_2_] *ϵ*HO_2_ + [CH_3_O_2_] *ϵ*CH_3_O_2_ = constant, applies. If *ϵ*HO_2_ and/or *ϵ*CH_3_O_2_ are not simultaneously adjusted a large bias occurs (columns 6 and 7, Table 2). In the next section the consequences of fixing *ϵ*HO_2_ and/or *ϵ*CH_3_O_2_ at “imperfect” values are explored. In such cases the constraints of Eq. (2) must be take into account explicitly to properly set [HO_2_]_0_ and [CH_3_O_2_]_0._

A relatively small bias in *k*_3_ develops regardless of which parameter is fixed at an imperfect value. If we were to conclude our error analysis at this point and analyze real “laboratory” data at the 1% *n/s* level we would expect an absolute error of about 0.19, Table 1, due entirely to the random noise and independent of any bias. If the initial concentrations of HO_2_ and CH_3_O_2_ were uncertain to the extent of 10% we would expect a small bias of ±0.07 (in *k*_3_) due to CH_3_O_2_ (compare *k*_3_ in columns 6 and 8, Table 2, with 2.90) and a bias of ±0.23 due to HO_2_ (compare columns 9 and 10, Table 2, with 2.90). Assuming the random errors and the biases can be combined through vector addition the combined error in *k*_3_ would be: *σ*^2^(*k*_3_) = (0.19)^2^ + (0.07)^2^ + (0.23)^2^ = (0.31)^2^. Additionally if *k*_2_ were fixed and its estimated error were also at the 10% level, the term (0.44)^2^ would have to be added. If *k*_1_ were fixed, a 10% estimated error in this parameter, according to Table 2, would require adding the additional term (0.135)^2^ in order to obtain the combined error in *σ*^2^(*k*_3_)= (0.55)^2^ (an error of 19% in (*k*_3_). This assumes that the errors in the fixed parameters can be properly estimated and they are not correlated. An error in both [HO_2_]_0_ and [CH_3_O_2_]_0_ with no error in *ϵ*HO_2_ and *ϵ*CH_3_O_2_ was assumed. The error estimates are associated with the product of the absorption coefficient and the initial concentration for each species. The errors in the initial concentration and the ϵ (for each species) are in fact distributed.

The principal conclusions are, i) that when one or more parameters are fixed, the bias on *k*_3_ (the parameter of interest) that each fixed parameter produces must be determined, and ii) If the biases are small, as in the present case, errors given by the variance-covariance matrix are still valid. They encompass the correct or expected results (column 11, Table 2). If results fall outside of the error estimates obtained from the variance-covariance matrix the composite error can be obtained by vector addition of the random error, due to noise, and the bias engendered by fixing a parameter.

### 4.3. Fixing *ϵ*HO_2_ and/or *ϵ*CH_3_O_2_ at “Non-Perfect” Values

We continue the analysis of Table 2 by fixing the two important absorption coefficients at “imperfect” values. This is equivalent to using a erroneous value for one or both of these coefficients in the analysis of real laboratory data. Here we are both compliant and noncompliant with the constraints of Eq. (2). Thus, *ϵ*HO_2_ and *ϵ*CH_3_O_2_ are individually or simultaneously varied and the initial concentrations [HO_2_]_0_ and [CH_3_O_2_]_0_ are fixed at values dictated by Eq. (2), or are fixed at values given in column 1 of Table 3. The results show that non-adherence to the conditional constraints of Eq. (2), will not adversely affect the bias on *k*_3_ (compare columns 2 and 3, Table 3) if *ϵ*HO_2_ is fixed (10%) off its “perfect” value. A large bias does occur, however, if *ϵ*CH_3_O_2_ is fixed ±10% off from its “perfect” value and the initial concentrations [HO_2_]_0_ and [CH_3_O_2_]_0_ are not initialized in accord with Eq. (2) (see results of columns 4 and 5). Columns 9 and 10 show a significant bias on *k*_3_ if eHO_2_ is set ±10% and *ϵ*CH_3_O_2_ is set ±10% off from their perfect values with both offset in the same direction.

### 4.4 Optimum [HO_2_]_0_/[CH_3_O_2_]_0_ Condition for Obtaining *k*_3_ at 250 nm

We explore here how the absolute error in *k*_3_ varies with the ratio [HO_2_]_0_/[CH_3_O_2_]_0._ It may be guessed that *k*_3_ is best-fit under conditions where the cross-combination product builds up to its largest possible value, but at the same time it is desired to maximize the absorption signal. The noise level in the absorption signal is a function of the intensity of the analysis light beam (the signal to noise ratio improves linearly with the absorption signal). It is not known *a priori* which of these two optimization conditions most influences the minimization of error in *k*_3_ or whether other factors also contribute.

**Fig. 2:**
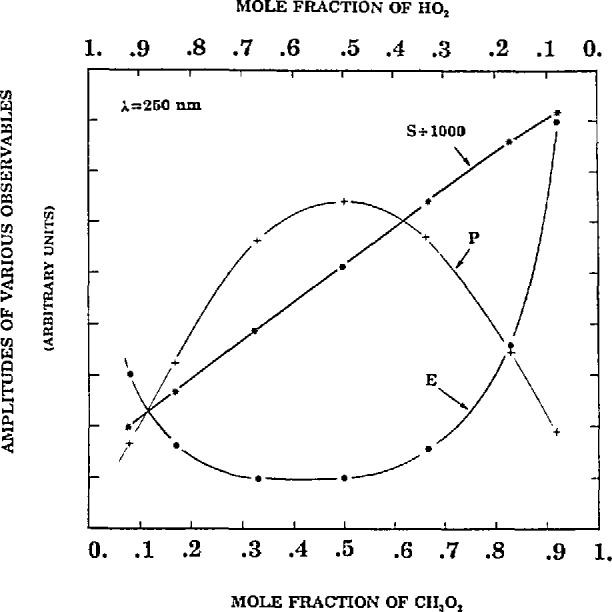
Absolute error in *k*_3_, *E*; Absorption signal at 250 nm, *S*; and cross-combination product plotted vs mole fractions of [CH_3_O_2_]_0_ and [HO_2_]_0_. Parameters same as in column 1, Table 1; and [CH_3_O_2_]_0_ + [HO_2_]_0_ = 1.2.

Figure 2 shows the result of the error analysis obtained by varying the ratio [HO_2_]_0_/[CH_3_O_2_]_0_. The absolute error in *k*_3_ is obtained by first preparing a “synthetic” laboratory data file appropriate to the initial concentrations at the specified noise level, *σ* =3.66 corresponding to a *n*/s=0.01, with all of the other parameters given under Eq. (1). Then *k*_3_ is adjusted (best-fit) fixing all other parameters at their “perfect” values.

The total absorption signal (*S*), the final cross-combination product (*P_t= ∞_*) and the error in determining *k*_3_ (E) are plotted in Fig. 2. Since (*P*) maximizes at about a 50/50 composition ratio of [CH_3_O_2_]_0_/[HO_2_]_0_ and (5) maximizes well to the right (side of the figure) a minimum in the error in *k*_3_ would be expected at a composition ratio greater than 50/50. In fact a minimum is obtained at about 40/60. The prediction indicates that factors other than the maximum signal and cross-combination product enter into the optimization analysis. The minimum error in *k*_3_ occurs over a fairly extensive range of initial concentrations. Clearly the optimization of the composition condition must be made by minimizing the error in *k*_3_.

## 5. Optimization of [HO_2_]_0_/[CH_3_O_2_]_0_ to Obtain Several Parameters at 215 nm

**Fig. 3:**
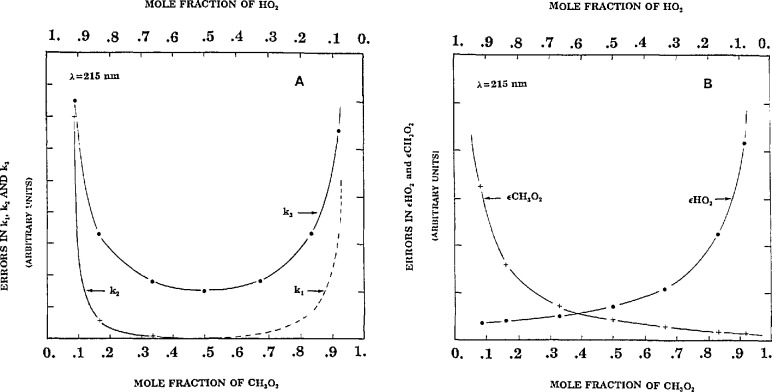
Absolute errors in *k*_1_, *k*_2_, and k_3_ vs mole fractions of [CH_3_O_2_]_0_ and [HO_2_]_0_ at 215 nm. Rate parameters same as in column 1, Table 1; absorption coefficients (for λ = 215.), Conditions as in Eq. (1); [CH_3_O_2_]_0_ + [HO_2_]_0_ = 1.2. B: Absolute errors in *ϵ*CH_3_O_2_ and *ϵ*HO_2_ are similarly plotted.

We extend the above analysis by optimizing [HO_2_]_0_/[CH_3_O_2_]_0_ with respect to several of the parameters, taken one at a time. The experiment is at 215 nm and the appropriate parameters of Eq. (1) are used. The results of the analysis are shown in Fig. 3. It is noted that the analysis displayed in Fig. 2 (experiment at λ =250 nm) and 3 (λ =215 nm) can be directly compared with respect to the magnitude of the error on *k*_3_, common to both figures.

The optimization conditions for obtaining the parameters *k*_1_ and *k*_2_ (also *ϵ*HO_2_ and *ϵ*CH_3_O_2_) are obvious but it is instructive to examine the detailed shapes of the curves. Clearly for the self reactions the error in the determination of the k’s is smallest when the associated radical concentration is dominant. The optimum ratio [CH_3_O_2_]_0_/[HO_2_]_0_ = 50/50 producing the least error in *k*_3_ is somewhat different from that found in the above case ([CH_3_O_2_]_0_/[HO_2_]_0_ = 40/60) probably because at 215 nm ϵHO_2_ is comparable in magnitude to ϵCH_3_O_2_. Again there is no way of guessing the outcome.

If the ratio of the two initial concentrations were to be optimized to obtain the least error in several parameters *combined*, a 50/50 mixture of [HO_2_]_0_ and [CH_3_O_2_]_0_ would probably be the best compromise condition. We might expect to be able to solve for more than four parameters uniquely, as found previously using a nonoptimum [HO_2_]_0_/[CH_3_O_2_]_0_ ratio. However, it is found that only four parameters can be assessed, regardless of how small the noise level is set. This means that some combination of parameters yield fits that are about as good as other combinations. The system must be further constrained by fixing some of them. Setting [HO_2_]_0_/[CH_3_O_2_]_0_ significantly off center ([HO_2_]/[CH_3_O_2_]_0_ = 50/50, in Fig. 3) does have the expected effect of further restricting the number of parameters that can be varied simultaneously.

The combined error estimate in *k*_3_ for a 50/50 mix of [HO_2_]_0_/[CH_3_O_2_]_0_ (the optimum mixture) is recalculated. This is done as previously by determining the bias produced in *k*_3_ by assuming a 10% error in both *ϵ*HO_2_ and *ϵ*CH_3_O_2_ instead of in [HO_2_]_0_ and [CH_3_O_2_]_0_ We also include the bias produced by 10% errors in *k*_1_ and *k*_2_, as well as the random error produced by a *n/s* = 0.01. A somewhat lower error estimate is obtained, *σ* = ±0.30 (an error of ±10%) as compared to *σ* = 0.55 (an error of 19%) determined in Sec. 4.2 for nonoptimum mixtures. This error was evaluated by fixing all parameters and solving for *k*_3_, thereby allowing a direct comparison with the estimated error of the previous Ref. [5]. Reevaluating the error in *k*_3_ obtained in Ref. [5] where the percent error in both *ϵ*HO_2_ and *ϵ*CH_3_O_2_ were chosen to be 30%, we find that at a 10% level and including the additional bias due to 10% errors in *k*_1_ and *k*_2_, we arrive at an error of about 20% in *k*_3_, similar to the range of values obtained here. Even at the 2*σ* level, this error does not completely encompass the range of values reported in the literature [9]. Nevertheless, a proper accounting for each study would have to be performed separately under their unique set of boundary conditions, to ascertain whether the remaining differences are attributable to larger errors in the input parameters or other factors (such as secondary chemistry).

## 6. Conclusions

We have shown that the errors returned by the variance covariance matrix are reliable when certain parameters are adjusted and “perfect” values are used for the fixed parameters. Systematic errors introduced by employing “imperfect” values for the fixed parameters can be evaluated (and corrected for) if a small bias results in the adjusted parameter-of-interest (here *k*_3_). The use of a generic least squares analysis program applicable to all kinetic mechanisms has been described, which can be used to optimize the conditions of an experiment. Monte carlo simulations are employed and the assumption has been made that they are reliable surrogates to real “laboratory” data.

## Figures and Tables

**Table 1:** Different analysis fixing various parameters at their “perfect” values and adjusting those remaining

Parameters	Values	*n/s* = 0.01	*n/s* = 0.01	*n/s* = 0.01	*n/s* = 0.01	*n/s* = 0.01	*n/s* = 0.01	*n/s* = 0.01	*n/s* = 0.01	*n/s* = 0.05	*n/s* = 0.01	*n/s* = 0.05	*n/s* = 0.01	*n/s* = 0.05

0	1	2^a^	3^a^	4^a^	5^a^	6	7^a^	8^a^	9	10	11	12	13	14
*ϵ*HO_2_	60	57.2 ± 10	*F*	12.8 ± 84.	161 ± 178.	*F*	*F*	*F*	54.3 ± 11.3	38.1 ± 56.3	*F*	*F*	*F*	*F*
*ϵ*CH_3_O_2_	365	*F*	364 ± 3.5	380 ± 27	330 ± 63.	365 ± 3.2	*F* *F*	*F* *F*	367 ± 2.7	372 ± 10.9	366 ± 2.2	369. ± 9.3	*F* *F*	*F* *F*
*ϵ*H_2_O_2_	8.3	*F*	*F*	*F*	*F*	*F*	*F*	*F* = 0.0	*F*	*F*	*F*	*F*	*F*	*F*
*ϵ*CH_3_O_2_H	3.98	*F*	*F*	*F*	*F*	*F*	*F*	*F* = 0.0	*F*	*F*	*F*	*F*	*F*	*F*
*k*_i_	1.86	1.25 ± 0.05	1.26 ± 1.03	0.97 ± 1.5	*F*	*F*	1.38 ± 0.97	1.31 ± 0.94	*F*	*F*	*F*	*F*	*F*	*F*
*k*_2_	0.36	0.34 ± 0.02	0.34 ± 0.03	*F*	0.31 ± 0.09	0.36 ± 0.01	0.35 ± 0.02	0.34 ± 0.02	*F*	*F*	*F*	*F*	*F*	*F*
*k*_3_	2.90	3.02 ± 0.17	3.01 ± 0.20	3.18 ± 0.23	2.59 ± 0.78	3.05 ± 0.20	3.05 ± 0.12	3.05 ± 0.12	3.08 ± 0.19	3.82 ± 1.12	3.08 ± 0.20	3.80 ± 1.07	3.00 ± 0.10	3.52 ± 0.63
[CH_3_O_2_]_0_	0.88	*F*	*F*	*F*	*F*	*F*	*F*	*F*	*F*	*F*	*F*	*F*	*F*	*F*
[HO_2_]_0_	0.30	*F*	*F*	*F*	*F*	*F*	*F*	*F*	*F*	*F*	*F*	*F*	*F*	*F*

a The combination of parameters adjusted in this column cannot be uniquely determined if *n/s* = 0.05.

**Table 2:** Bias produced on adjusted parameters by fixing selected parameters at “non-perfect” values

Parameters	Values	*n/s* = 0.0	*n/s* = 0.0	*n/s* = 0.0	*n/s* = 0.0	*n/s* = 0.0	*n/s* = 0.0	*n/s* = 0.0	*n/s* = 0.0	*n/s* = 0.0	*n/s* = 0.01

0	1	2	3	4	5	6	7	8	9	10	11
*ϵ*HO_2_	60	91.7	29.4	49.	163.	*F*	F	F	55.	66.	63. ± 11.0
*ϵ*CH_3_O_2_	365	354.	375.	402.	330.	321.	F	407.	*F*	F	F
*ϵ*H_2_O_2_	8.3	*F*	*F*	*F*	*F*	*F*	*F*	F	*F*	F	F
*ϵ*CH_3_O_2_H	3.98	*F*	*F*	*F*	*F*	*F*	*F*	*F*	*F*	F	F
*k*_1_	1.86	*F* = 2.05	*F* = 1.67	1.18	2.43	1.27	5.44	2.30	2.06	1.52	0.81 ±
*k*_2_	0.36	0.35	0.37	*F* = 0.40	*F* = 0.32	0.32	0.44	0.39	0.35	0.37	0.35 ± 0.02
*k*_3_	2.90	2.76	3.03	3.31	2.43	2.82	7.40	2.96	2.69	3.15	3.28 ± 0.19
[CH_3_O_2_]_0_	0.88	*F*	*F*	*F*	*F*	*F* = 1.0	*F* = 1.0	*F* = 0.79	*F*	F	F
[HO_2_]_0_	0.30	*F*	*F*	*F*	*F*	F	F	F	*F* = 0.33	*F* = 0.27	*F* = 0.27

**Table 3:** Bias produced on adjusted parameters by fixing absorption coefficients and concentrations at “non-perfeet” values while maintaining constraints of Eq. (2)

Parameters	Values	*n/s* = 0.0	*n/s* = 0.0	*n/s* = 0.0	*n/s* = 0.0	*n/s* 0.0	*n/s* = 0.0	*n/s* = 0.0	*n/s* = 0.0	*n/s* = 0.0

0	1	2	3	4	5	6	7	8	9	10
*ϵ*HO_2_	60	*F* = 66.	*F* = 66.	*F*	*F*	*F*	*F*	*F* = 54.	*F* = 54.	*F* = 66.
ϵCH_3_O_2_	365	*F*	*F*	*F* = 400.	*F* = 400.	*F* = 474.	*F* = 328.	*F*	*F* = 328.	*F* = 400.
ϵH_2_O_2_	8.3	*F*	*F*	*F*	*F*	*F*	*F*	*F*	*F*	*F*
ϵCH_3_O_2_H	3.98	*F*	*F*	*F*	*F*	*F*	*F*	*F*	*F*	*F*
*k*_1_	1.86	*F*	*F*	*F*	*F*	*F*	*F*	*F*	*F*	*F*
*k*_2_	0.36	*F*	*F*	*F*	*F*	*F*	*F*	*F*	*F*	*F*
*k*_3_	2.90	2.71	2.96	2.20	5.70	1.80	4.90	3.20	5.00	2.00
[CH_3_O_2_]_0_	0.88	*F* = 0.87	*F*	*F* = 0.79	*F*	*F* = 0.65	*F* = 1.00	*F* = 0.89	*F* = 1.01	*F* = 0.78
[HO_2_]_0_	0.30	*F* = 0.31	*F*	*F* = 0.39	*F*	*F* = 0.53	*F* = 0.18	*F* = 0.29	*F* = 0.17	*F* = 0.40

## 7. Appendix A. Unique Solution

It is often difficult to ascertain if the parameters that are searched for (adjusted) are uniquely found. We define a unique fit purely operationally. If one or more parameters are initialized (guessed at) at different values we should arrive at the same ultimate best fit of the parameters (after several iterations). If this happens we define the fit to be unique. A second criterion involves employing two entirely different versions of Acufit. One program employs the general method outlined by Went-worth [11] which elaborates on Deming’s text [2] and involves using the SVD method of matrix solution [12]. The second program employs the more recent Levenberg-Marquardt method [12] and involves using the Gauss-Jordan matrix solution. Both programs yield exactly the same values for the adjusted parameters (and variance-covariance matrix) when a unique solution is possible. They yield somewhat different results if a solution is not obtained; then both result in unrealistic variance-co-variance matrices, the first program gives some (or all) matrix entries that are far too small, the other gives some entries that are much too high. Only when both programs yield the same answers do we operationally define that “the” solution exists. So far we have not encountered a problem where one program succeeds in finding a “unique” solution while the other does not.
